# Spike Activator 1, Encoding a bHLH, Mediates Axillary Bud Development and Spike Initiation in *Phalaenopsis aphrodite*

**DOI:** 10.3390/ijms20215406

**Published:** 2019-10-30

**Authors:** Yi-Jyun Lin, Min-Jeng Li, Hung-Chien Hsing, Tien-Kuan Chen, Ting-Ting Yang, Swee-Suak Ko

**Affiliations:** 1 Academia Sinica Biotechnology Center in Southern Taiwan, Tainan 741, Taiwan; hbabyi@gate.sinica.edu.tw (Y.-J.L.); lmz8332@yahoo.com.tw (M.-J.L.); sanny618@gmail.com (H.-C.H.); s4life2010@gmail.com (T.-K.C.); tty0318@gmail.com (T.-T.Y.); 2 Agricultural Biotechnology Research Center, Academia Sinica, Taipei 115, Taiwan

**Keywords:** moth orchid, axillary bud, spike induction, spiking, gene regulation, bHLH, FT, expansin

## Abstract

Double-spikes *Phalaenopsis* orchids have greater market value than those with single-spike. In this study, a gene designated as *Spike Activator 1* (*SPK1*), which encodes a basic helix-loop-helix (bHLH) transcription factor, was isolated and characterized from *Phalaenopsis aphrodite* (moth orchid). *SPK1* was highly expressed in the meristematic tissues. In the axillary bud, *SPK1* was highly upregulated by a moderately low temperature of 20 °C but downregulated by a spike inhibition temperature of 30 °C. SPK1 protein is localized in the nucleus. Another bHLH, *bHLH35*, which is also highly expressed in young tissues in the same way as *SPK1* was also identified. In contrast to *SPK1*, *bHLH35* transcripts are downregulated at 20 °C but upregulated at 30 °C. Bimolecular florescence complementation assay and yeast two-hybrid assays indicated that SPK1 interacts with bHLH35 and forms a heterodimer. Virus-induced gene silencing (VIGS) showed that 7 out of 15 vector control plants produced double spikes but that only 1 out of 15 VIGS-*spk1* plants produced double spikes. RT-qPCR results indicated that VIGS-*spk1* downregulated gene expression levels of *SPK1*, *FT*, *CYCB*, and *EXPA8*. Overall, we propose that SPK1 plays an essential role in early axillary bud development and spike initiation of *P. aphrodite*.

## 1. Introduction

*Phalaenopsis aphrodite* (moth orchid) is one of the most important export ornamental plants in the world. The potted orchid and cut-flower orchid market is flourishing, and it is a growing industry. Floral spikes of *Phalaenopsis* orchids are initiated from axillary buds at the leaf base. After a period of moderately low-temperature induction for one and a half months, axillary buds enlarge, break dormancy, and protrude from the leaf base to develop into young floral spikes. 

Temperature is a critical factor in spike induction of *Phalaenopsis* orchids. Some researchers recommend a day/night temperature fluctuation of 25 °C/20 °C or 20 °C/15 °C [1]. However, some indicated that *Phalaenopsis* orchid can initiate spikes at constant 14 to 23 °C or fluctuating day/night temperatures of 20 °C/14 °C or 23 °C/17 °C. It has also been found that orchids produced more spikes under constant 14 °C and 17 °C. Therefore, researchers concluded that day/night temperature fluctuation is not necessary for spike initiation [2]. Conversely, *Phal.* orchid spiking was significantly inhibited when it was grown at a temperature higher than 28 °C [3,4]. In addition to temperature, low light resulted in low photosynthesis rate and delayed spiking in moth orchid [5]. Once orchid spiking was initiated, the plants took 10 to 15 weeks to flower at 20 to 23 °C [2,6].

FLOWERING LOCUS T (FT) is a flowering marker in many plant species. In the shoot apical meristem (SAM), FT interacts with FLOWERING LOCUS D (FD) to promote flowering and the activation of floral meristem identity genes. Therefore, FT may represent a long-distance signal in flowering [7]. Some genes controlling flowering in orchid have been reported recently. *Phalaenopsis* orchid FT interacting with FD might regulate downstream genes such as *AP1*, *SOC1*, and *Leafy* [8]. FD and FT are interdependent partners through protein interaction and act at the shoot apex to promote floral transition and to initiate floral development through transcriptional activation of a floral meristem identity gene, *APETALA1* (*AP1*). FT protein is transported to the SAM via the phloem [9]. 

Cell-cycle control is important for plant growth and development. Cyclins activate the kinase activity of the cyclin-dependent kinases (CDKs) and control timely cell-cycle processes [10]. CYCB gene family members are preferentially accumulated during S, G2, and M phases and are often used as markers for dividing cells [11]. *Phal.* orchid CYCB1;1 is highly expressed in the developing embryos and in the meristem tissues of the protocorm [10]. Also, CYCB1;1 is expressed more abundantly in the 3rd axillary bud than the 2nd axillary bud, as revealed by In Situ Hybridization (ISH) [12]. Study of the relationship between endodormancy, FT, and cell cycle genes indicated that FT plays a key role in regulating cell-cycle genes transcriptionally [13]. 

Expansins are classified by sequence-based phylogeny into two major families named EXPA (α-expansins) and EXPB (β-expansins) [14]. Expansins are cell-wall-loosening proteins that mediate acid-induced growth by catalyzing loosening of plant cell walls [15]. During growth, plant cells secrete expansins to loosen wall polysaccharides and to permit cell enlargement [16]. 

MYB is the largest, and basic helix-loop-helix (bHLH) is the second largest gene family. The MYB and bHLH families also interact with a number of other regulatory proteins, forming complexes that either activate or repress the expression of sets of target genes [17]. bHLH transcription factors (TFs) are involved in numerous biological processes in plants including responses to cold, light, and hormones; epidermal cell fate determination; and development of roots and flowers [18,19,20,21,22,23,24,25,26]. LAX PANICLE1 Protein (LAX1), a bHLH, is required for axillary meristem formation [27]. A bHLH complex of LHW-T5L1 activates vascular cell division via cytokinin action in the apical meristem of the root [28]. 

Little work has been done to address the gene regulatory pathways of axillary bud and spike development in orchid. To understand the physiological, cellular, and molecular processes of floral spike initiation in moth orchid, in this study, we carried out extensive experiments and isolated a novel bHLH, *Spike Activator 1* (*SPK1*), which is highly expressed in the meristematic tissues of orchid and upregulated by moderately low temperature for spiking. SPK1 protein can form a homodimer and heterodimer with bHLH35. Virus-induced gene silencing (VIGS) experiment demonstrated that SPK1 is an important regulator mediating axillary bud development, spike initiation, and reproductive organ development in *Phal.* orchid. 

## 2. Results

### 2.1. Morphology of Axillary Bud Development after Spike Induction of Phal. Orchid

**Figure 1:**
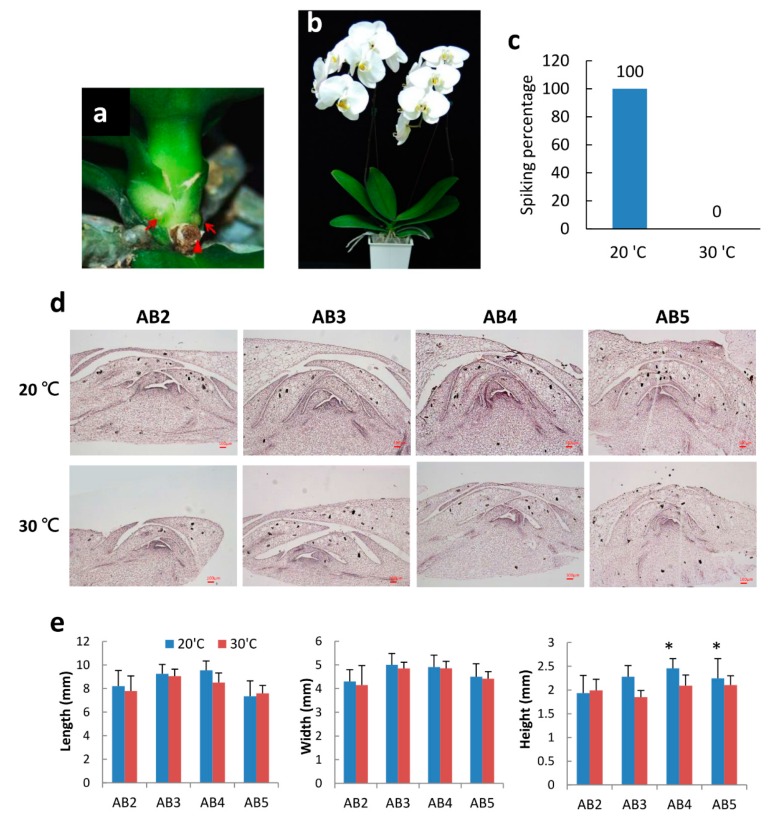
Effect of temperature on the growth of axillary buds of *Phalaenopsis* orchids: (**a**) Axillary buds grew in the leaf base. (**b**) Blooming of *Phal.* orchid at 3.5 months after spiking. (**c**) Moderate low temperature (20 °C) enhanced orchid spiking. (**d**) Morphology of axillary buds in different leaves of *Phal*. orchids after treated at 20 °C and 30 °C for 2 weeks. AB2, young axillary bud in the 2nd leaf; AB5, older axillary bud in the 5th leaf. Bar, 100 μm. (**e**) Size of axillary buds including length, width, and height. Error bars indicate the SD of 7 plants. Asterisks indicate significant difference in 20 °C compare to 30 °C (*p* < 0.05, Student’s *t*-test).

Monopodial *Phal.* orchid has an upright main stem with several leaves growing in the alternate leaf positions. The axillary bud is a small organ embedded at the leaf base (Figure 1a). After axillary bud maturation and breaking dormancy, floral spikes will initiate and protrude from the leaf base. When a young spike (>2 cm in length) is observed, the spike development stage is defined as “spiking”. The process of moderately low-temperature treatment to induce spiking is called “spike induction”. Young spikes will then elongate to form a flower stalk and to develop flower buds, and blooming takes another 3 to 4 months after spiking (Figure 1b). *Phal*. orchid spiking is temperature and genetic-factor dependent [4]. In this study, we used commercial big white orchid cultivar “N2K01” mature 3.5” potted plants and compared spiking and non-spiking temperatures of 20 °C and 30 °C, respectively. After treatment for 2 months at 20 °C, all plants can produce one to two young spikes but absolutely no spiking was observed after 30 °C treatment (Figure 1c). Hence, we confirmed spike induction at 20 °C is appropriate for this cultivar.

To do a detailed observation of axillary bud development during the spike induction process, orchid plants were kept at 20 °C (spiking condition) and 30 °C (non-spiking condition) for two weeks. Then, axillary bud samples were collected from top to bottom from the 2nd leaf (denoted AB2) to the 5th leaf (denoted AB5) fixed in PFA buffer, dehydrated with serial ethanol, and embedded in a paraffin block, and tissue was sliced vertically to 10-µm thickness. After serial dewaxing and rehydration processes, issues were stained with hematoxylin. Tissue section images were taken under microscope. Enlargement of axillary bud size was found in the axillary bud of AB3 to AB5 after 20 °C treatment. Outer scale number was increased, and the meristematic tissue showed enlargement (Figure 1d, top panel), whereas, at 30 °C, the growth of the young axillary buds (AB2 and AB3) was strongly inhibited and showed smaller size AB4 and AB5 (Figure 1d, bottom panel) compared to those at 20 °C. Quantitative measurement of the length, width, and height of the axillary bud size was carried out at two weeks after treatment. Results indicated that AB3 and AB4 had longer and wider axillary buds, indicating that these axillary buds were more mature than AB2. After 20 °C spike induction for two weeks, axillary buds of AB3, AB4, and AB5 grew higher and have large meristems. However, small-size dormant axillary buds were found after 30 °C treatment (Figure 1e). Normally, N2K01 mature plants can produce one to two spikes after spike induction. The position of the floral stalk will come from the 3rd to 5th leaf positions depending on the maturation of the axillary buds. It is consistent with our finding that AB3 to AB5 are larger and more mature than young AB2. Besides bud maturation, appropriate low temperature can induce dormancy breaking and turn on spike initiation in *Phal.* orchid. In this study, all N2K01 plants exhibited spiking after 20 °C treatment; however, absolutely no spiking was found under high temperature of 30 °C (Figure 1C).

### 2.2. Identification of a bHLH Gene Responding to Spiking 

**Figure 2:**
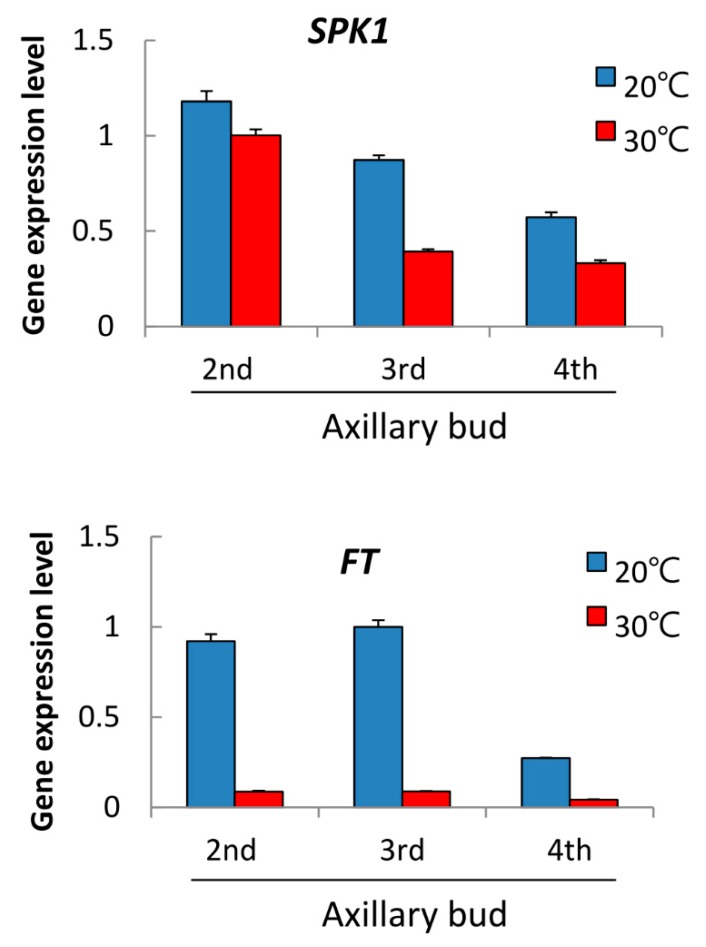
*SPK1* responds to temperature treatments: Axillary buds of *Phal.* orchid after treatment at 20 °C and 30 °C for 2 weeks were collected, and gene expression patterns of *SPK1* (**a**) and *FT* (**b**) were detected. Relative gene expression was quantified by real-time PCR and normalized to *Ubiquitin*. Data are presented as mean ± SD (*n* = 3).

In order to understand gene profiling during orchid spiking, we performed RNAseq using the 4th axillary bud after treatment at 20 °C (spiking condition) and 30 °C (non-spiking condition) for two weeks (data not shown). Our team is interested in identifying the gene function of bHLH TFs, and we found that a *bHLH* gene was significantly upregulated at 20 °C but inhibited at 30 °C, which piqued our interest to do further study. First of all, we performed RT-qPCR on this bHLH candidate in various axillary buds after 20 °C and 30 °C treatment for two weeks, along with *FT,* a flowering marker gene. Results indicated that this target *bHLH* gene was upregulated at 20 °C but inhibited at 30 °C. At 20 °C, the candidate *bHLH* gene was highly expressed in AB2, was second most highly expressed in AB3, and decreased in AB4. Similarly, at 30 °C, the candidate *bHLH* gene was more significantly expressed in the young AB2 than AB3 and AB4 (Figure 2a). As a flowering marker, *FT* was highly expressed after 20 °C spike induction, and abundant transcripts were found in the axillary buds of AB2 and AB3, whilst *FT* mRNA was inhibited at 30 °C (Figure 2b). As this bHLH candidate may be associated with spiking, we named it “Spike Activator 1 (SPK1)” in this study. The experiments were repeated twice, and the results were consistent. Therefore, we were interested in conducting a detailed study on the biological function of SPK1.

### 2.3. Isolation of SPK1 Full-Length cDNA and Its Protein Subcellular Localization in the Nucleus 

SPK1 was blasted to PATC136668 in the Orchidstra 2.0 database [29]. It is annotated as “Similar to XP_009403078, PREDICTED: transcription factor bHLH94-like” in the database. Full-length cDNAs of SPK1 were cloned from *Phal. aphrodite* using the RACE PCR method. The *SPK1* cDNA was 1344 bp in length with a 912-bp coding sequence. The 5′ UTR length was 213 bp, and the 3′ UTR was 219 bp. Sequence data of SPK1 was deposited with the GenBank data libraries under accession number MN481612.

**Figure 3:**
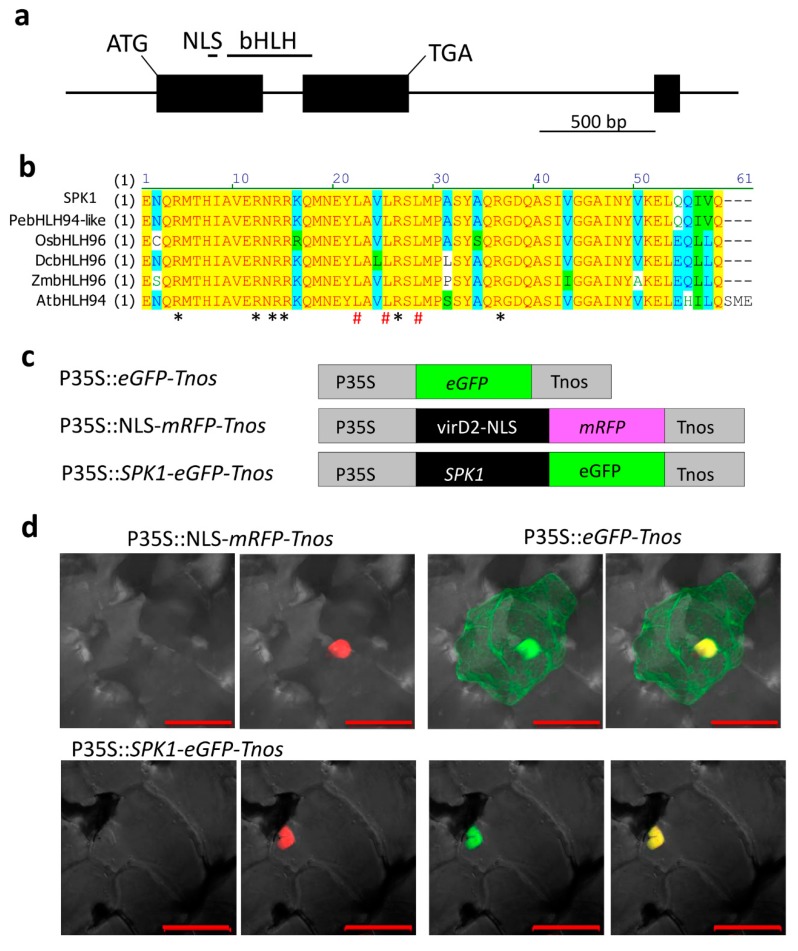
Gene structure, multiple alignment, and subcellular localization of SPK1: (**a**) Gene structure of *SPK1*. Lines, introns. Black boxes, exons. Bar, 500 bp. (**b**) SPK1 basic helix-loop-helix (bHLH) conserve domain multiple alignment. *At, Arabidopsis_thaliana; Dc., Dendrobium catenatum; Os., Oryza sativa; Pe., Phalaenopsis equestris; Zm., Zea_mays.* Asterisks indicate the conserved basic amino acid Arg that is important for binding DNA. Pound signs indicate conserved Leu residues important for forming the α-helix. (**c**) Schemes of fusion constructs. P35S, cauliflower mosaic virus 35S promoter; Tnos, nopaline synthase gene terminator. The nuclear localization signal (NLS) domain of VirD2 fused with red fluorescent protein (mRFP) was used as the nuclear localized control. (**d**) Subcellular localization of SPK1 protein. Bars, 50 μm.

The gene structure of *SPK1*, shown in Figure 3a, indicates that *SPK1* contains a bHLH domain and a DNA-binding domain and contains a nuclear localization signal (NLS). The *SPK1* gene is predicted to encode a protein of 303 amino acids. Multiple alignment of the bHLH-conserved domain was carried out, and the results showed that SPK1 contains conserved basic amino acid Arg that is important for binding DNA and conserved Leu residues that are important for forming the α-helix for protein–protein interaction (Figure 3b). Since the bHLH proteins are characterized as TFs, we wondered whether SPK1 protein was localized in the nucleus. To verify the subcellular localization, we constructed a fusion gene of the green fluorescent protein gene (eGFP) and SPK1 under the control of the 35S promoter and the nos terminator for transient expression. As a positive control, the NLS sequence was also fused to the red fluorescent protein (RFP) gene using the same regulatory elements (Figure 3c). These constructs were bombarded into orchid petal cells. As expected, with the eGFP construct alone, the GFP signal was found constitutively expressed in the nucleus as well as in the cytoplasm. However, the SPK1:eGFP fusion protein and the positive control of NLS:RFP were exclusively located in the nucleus (Figure 3d). These results confirmed that SPK1 protein is localized in the nucleus.

### 2.4. Phylogenetic Tree 

**Figure 4:**
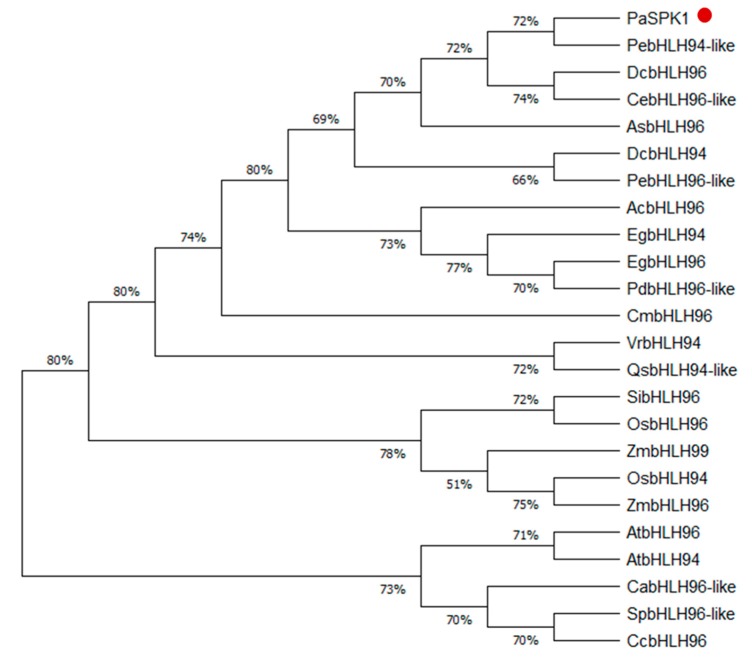
Phylogenetic analysis of the SPK1 full length protein: Amino-acid sequences of various species downloaded from the NCBI database, analyzed using Molecular Evolutionary Genetics Analysis version 10 (MEGA10), and constructed into a phylogenetic tree. The evolutionary history was inferred using the neighbor-joining method. The percentage of replicate trees in which the associated taxa clustered together in the bootstrap test (1000 replicates) are shown next to the branches. Boot-strapping values are indicated as percentages (when >50%) along the branches. The evolutionary distances were computed using the Poisson correction method and are in the units of the number of amino-acid substitutions per site. *Ac*, *Ananas comosus*; *As*, *Apostasia shenzhenica*; *At*, *Arabidopsis thaliana*; *Ca*, *Coffea arabica*; *Cc*, *Capsicum chinense*; *Ce*, *Cymbidium ensifolium*; *Cm*, *Cinnamomum micranthum*; *Dc*, *Dendrobium catenatum*; *Eg*, *Elaeis guineensis*; *Os*, *Oryza sativa*; *Pa*, *Phalaenopsis aphrodite*; *Pd*, *Phoenix dactylifera*; *Pe*, *Phalaenopsis equestris*; *Qs*, *Quercus suber*; *Si*, *Setaria italica*; *Sp*, *Solanum pennellii*; *Vr*, *Vigna radiata*; and *Zm*, *Zea mays*.

To determine the evolutionary relationship between SPK1 in *Phal. aphrodite* and other plant species, we constructed a phylogenetic tree for the SPK1 amino acid sequences from different species, including *Ananas comosus*, *Apostasia shenzhenica*, *Arabidopsis thaliana*, *Coffea arabica*, *Capsicum chinense*, *Cymbidium ensifolium*, *Cinnamomum micranthum*, *Dendrobium catenatum*, *Elaeis guineensis*, *Oryza sativa*, *Phalaenopsis aphrodite*, *Phoenix dactylifera*, *Phalaenopsis equestris*, *Quercus suber*, *Setaria italica*, *Solanum pennellii*, *Vigna radiata*, and *Zea mays*. A total of 24 full-length amino acid sequences have been used to produce a phylogenetic tree. The phylogenetic tree analysis data indicated that *Phal. aphrodite* SPK1 was closely related to other orchid species such as *Phal. equestris*, *Dendrobium catenatum*, and *Cymbidium ensifolium*. SPK1 has less similarity with AtbHLH94 (AT1G22490) (Figure 4).

### 2.5. Gene Expression Patterns of SPK1

**Figure 5:**
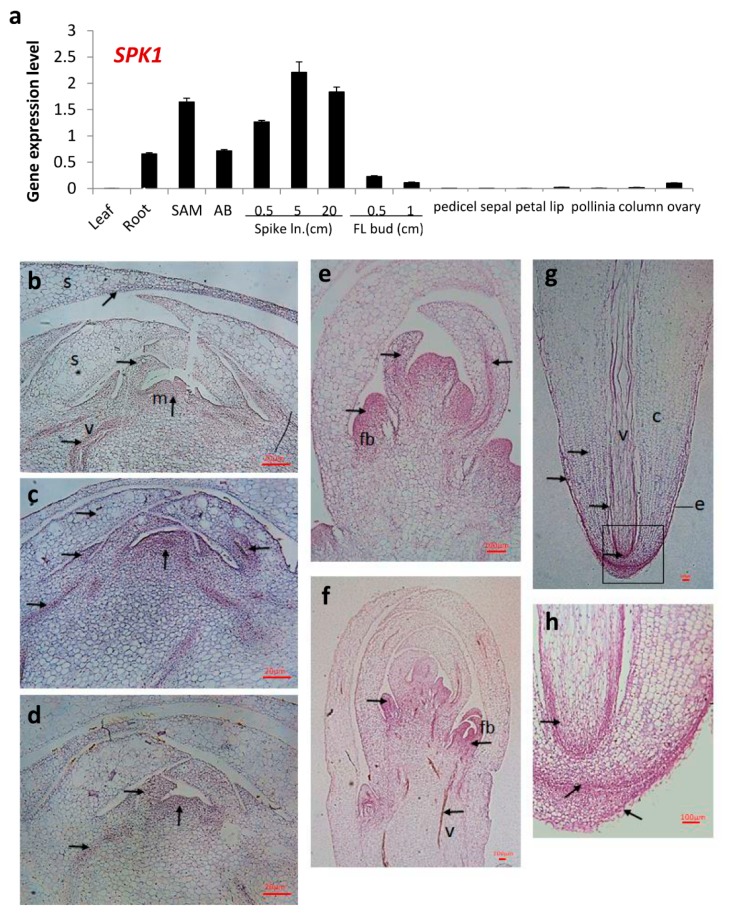
Gene expression patterns of *SPK1* in *Phal.* Orchid: (**a**) RT-qPCR showed deferential gene expression patterns of *SPK1* in various tissues of *Phal*. orchid. Relative gene expression was quantified by real-time PCR and normalized to *Ubiquitin*. Data are presented as mean + SD (*n* = 3). RNA in situ hybridization of *SPK1* antisense probe to various vertical sections for axillary buds of AB2 (**b**), AB3 (**c**), AB4 (**d**), young spike at 5 cm in length (**e**), spike at 10 cm in length (**f**), root tip (**g**), and magnified image of root tip (**h**). c, cortex; e, epidermis; m, meristem tissues; rc, root cap; s, scale; v, vascular bundle. The boxed area in (g) is magnified in (h). Arrows point to In Situ Hybridization (ISH)-positive signal. Scale bars: 20 µm (Figure 5b–c), 100 µm (Figure 5e–h).

To understand the gene expression pattern of *SPK1* in orchid, RNA samples from various tissues/organs of *Phal.* orchid were collected; the RNA was extracted, and quantitative RT-PCR (RT-qPCR) was carried out. It was found that *SPK1* gene was highly expressed in the young tissues such as root tip, shoot apex, axillary bud, and developing young spikes but that there was less expression in the leaf and mature flower organs (Figure 5a). In situ hybridization data revealed that *SPK1* mRNA was expressed in the young tissues of the axillary bud, developing young flower buds, and root tip (Figure 5b–h). Close-up observation of *SPK1* gene expression pattern in different ages of axillary buds indicated that the *SPK1* ISH-positive signal is expressed in the meristematic tissues, developing scales, and vascular bundles of young AB2 and AB3 (Figure 5b,c), with less ISH signal in the mature axillary bud tissue of AB4 (Figure 5d). In the top portion of developing young floral spikes, flower buds were initiated and *SPK1* was expressed broadly in flower bud primordia and more restricted in the meristem and vascular bundle (Figure 5e). Gradually, when the flower bud organ was differentiated, the *SPK1* RNA ISH signal was restricted in the meristematic tissues. Moreover, the vascular bundle of the floral spikes also had a strong ISH signal (Figure 5f). Similarly, the vascular bundle of the root also expressed *SPK1* gene. A strong ISH signal was observed in the root apical meristem and elongation zone (Figure 5g). Magnification of root image indicated that *SPK1* gene was expressed in the root cap, meristem, epidermis, and cortex; high in the phloem; and detectable in the xylem (Figure 5h). We also carried out ISH using *SPK1*-sense probe (as a negative control) in the relevant tissues of Figure 5. As shown in the Figure S1, nonspecific hybridization signal (i.e., background signal) was relatively weak using the sense probe of *SPK1*. To observe the morphology of tissues, all tissues used for the ISH experiment were also stained with hematoxylin and are shown in Figure S2.

### 2.6. bHLH35 Was Upregulated by High Temperature

**Figure 6:**
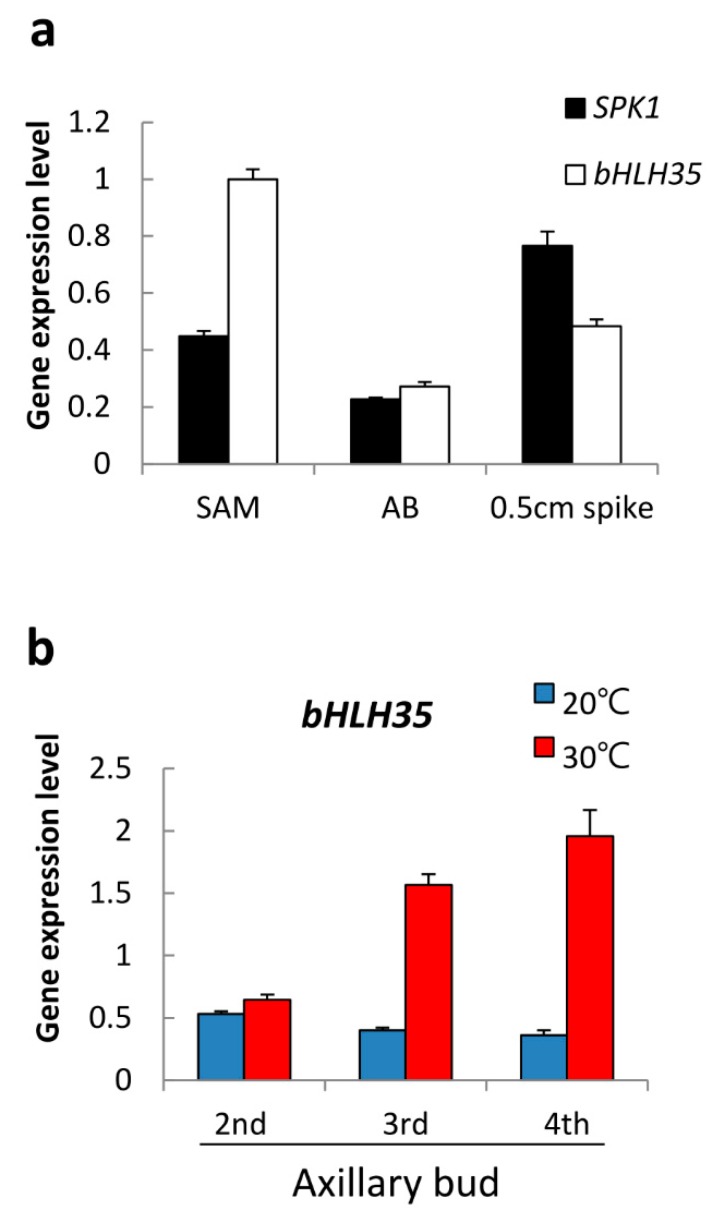
Gene expression patterns of *bHLH35* in *Phal.* Orchid: (**a**) *bHLH35* is highly expressed in the shoot apex (SAM), axillary bud (AB), and young spikes of 0.5 cm length. (**b**) High temperature at 30 °C upregulated *bHLH35* mRNA but it was downregulated at 20 °C. Relative gene expression was quantified by real-time PCR and normalized to *Ubiquitin*. Data are presented as mean ± SD (*n* = 3).

We also found another bHLH gene, *bHLH35* (PATC131347) from the RNAseq data. RT-qPCR showed that *bHLH35* was highly expressed in the young tissues of shoot apex, axillary bud, and 0.5 cm young spikes, as with *SPK1* (Figure 6a). However, *bHLH35* was expressed differently to *SPK1* in response to temperature stimuli. It was found that *bHLH35* was expressed at very low levels in the axillary buds after 20 °C treatment, but it was highly upregulated at 30 °C (Figure 6b). It is therefore postulated that bHLH35 may play an inhibitory role in spiking. 

### 2.7. SPK1 and bHLH35 Form a Heterodimer 

**Figure 7:**
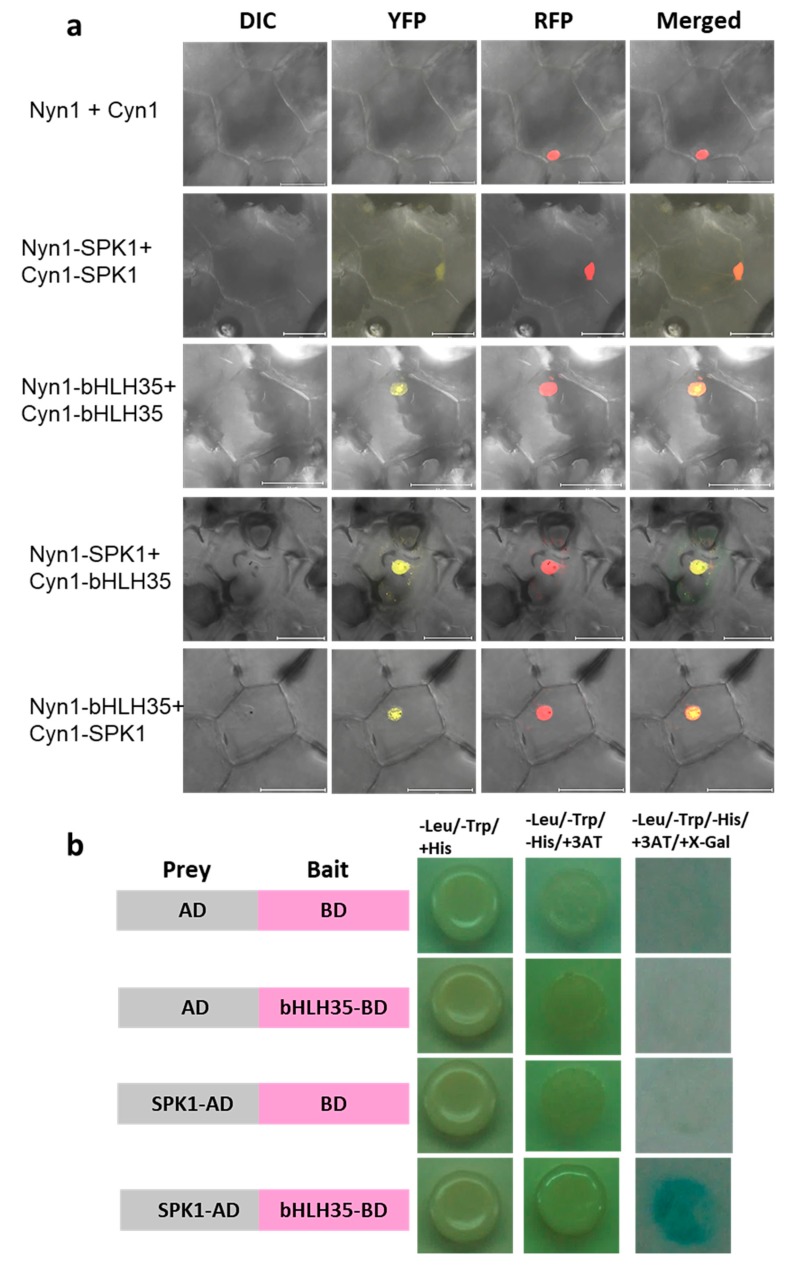
Protein–protein interaction between SPK1 and bHLH35: (**a**) bimolecular fluorescence complementation (BiFC) indicated SPK1 and bHLH35 can form a homodimer and that these two proteins can interact to form a heterodimer. The NLS domain of VirD2 fused with mRFP was used as the nuclear localized control. Bar: 50 µm. (**b**) yeast two-hybrid (Y2H) assays. Constructs expressing the full-length SPK1 were cloned into the prey vector pGADT7 (AD), and bHLH35 were introduced into the bait vector pGBKT7 (BD). Yeast strains were tested for growth in selective medium lacking Leu, Trp, and His and supplemented with 3-amino-1,2,4 triazole and X-Gal.

In general, bHLH TFs can form homodimers or heterodimers with other bHLH TFs and form a large variety of dimers to regulate distinct cellular and developmental processes. To know whether SPK1 and bHLH35 proteins formed a dimer, bimolecular fluorescence complementation (BiFC) assays were carried out. BiFC result showed that yellow fluorescent protein (YFP) signals were detected only in the nucleus of the orchid cells when co-expressing both NYN1-SPK1 and CYN1-SPK1, or co-expressing NYN1-bHLH35 and CYN1-bHLH35, indicating that SPK1 and bHLH35 itself can form a homodimer (Figure 7a). It was interesting to find that, in either co-expression of both NYN1-SPK1 and CYN1-bHLH35 or co-expression NYN1-bHLH35 and CYN1-SPK1, YFP signals were detected. These results confirmed that SPK1 and bHLH35 can form a heterodimer complex in the cell nucleus (Figure 7a). Next, we performed yeast two-hybrid (Y2H) analysis to determine whether bHLH35, as bait, interacts with the prey, SPK1. Our Y2H results indicated that the SPK1 and bHLH35 proteins are not self-activated. Only the yeast strains co-expressing both SPK1 and bHLH35 grew normally on stringent selection media (Figure 7b). Hence, BiFC and Y2H data confirmed that SPK1 can form homodimer; moreover, SPK1 can form a heterodimer complex with bHLH35.

### 2.8. Verification of SPK1 Function

**Figure 8:**
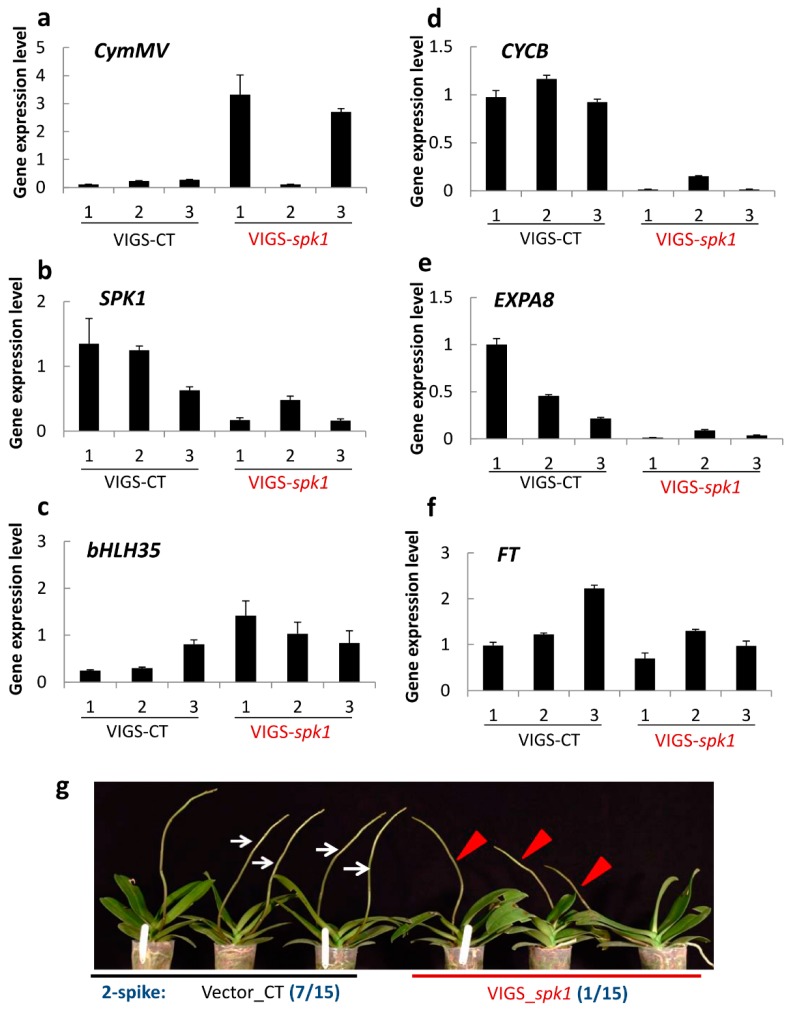
Validation of gene function using a Virus-induced gene silencing (VIGS) approach: VIGS of *SPK1* altered gene expression patterns of *CymMV* (**a**), *SPK1* (**b**), *bHLH35* (**c**), *CYCB* (**d**), *EXPA8* (**e**), and *FT* (**f**) in the 3rd axillary bud. Relative gene expression was quantified by real-time PCR and normalized to *Ubiquitin*. Data are presented as mean ± SD (*n* = 3). (**g**) VIGS-*spk1* affected double spiking rate of *Phal. aphrodite* cv. N2K01. Bar, SD of three replications. Arrows point to the double spikes; arrowhead points to the single spike. Photograph was taken at 3.5 months after spike induction.

In order to verify the biological function of SPK1 in orchid, we used a VIGS technique. The gene-specific region of *SPK1* was isolated and cloned into the pCymMV-Gateway vector [30]. The VIGS gene construction maps are shown in Figure S3. After agrobacterium infiltration, orchid plants were moved to a 20 °C growth chamber to induce spiking. At 6 weeks after VIGS treatment, axillary bud samples were collected, RNA was isolated, and RT-qPCR was performed. The results showed that after VIGS, virus *CymMV* gene was detected, indicating that VIGS-transmitted viral vectors are effective in orchid plants. *SPK1* gene expression was not altered in the AB3 of vector only control but showed sharp downregulation in the VIGS-*spk1* plant (Figure 8b). This showed that the gene silencing *SPK1* target gene is effective in the AB3 of VIGS-*spk1* plant. It was interesting to discover that, in the developing AB3 (the latter might develop to the 2nd floral spike), VIGS-*spk1* orchid also significantly downregulated *CYCB* and *EXPA8* but only slightly decreased *FT* (Figure 8d–f). Meanwhile, in the axillary bud of AB4 (more mature than AB3 but later will develop to the 1st floral spike), some VIGS-*spk1* plants have less gene silencing effects and expressed high *SPK1*, *CYCB*, *EXPA8*, and *FT* transcripts (Figure S4). These results suggest that AB4 is already mature before VIGS treatment and that the mature axillary buds immediately respond to 20 °C spike induction, turn on spiking signal, and upregulate spiking genes. It is interesting to find that *bHLH35* is upregulated whenever *SPK1* mRNA is downregulated in the VIGS-*spk1* plants (Figure 8C and Figure S4C). 

After VIGS for 3.5 months, spike number was recorded. Results showed that all orchid plants produced at least one floral stalk. It was found that all orchid plants can produce at least one spike after spike induction and that 7 out of 15 plants produced double spikes in the vector-only control, whilst a mere 1 out of 15 VIGS-*spk1* plants produced double spikes. Phenotype of spiking at 3.5 months after VIGS is shown in Figure 8g. The VIGS experiment clearly suggested that knockdown of *SPK1* tends to inhibit young axillary bud development and to reduce double spiking rate due to downregulation of *SPK1* and genes associated with spiking. 

## 3. Discussion 

### 3.1. Physiology of Spiking in Phalaenopsis Orchid 

Precise control of spiking is very important for the *Phalaenopsis* orchid industry and trading. A mature *Phalaenopsis* orchid at the five-leaf stage actually has five different sizes of axillary buds in the plant. Spike initiation first requires a mature axillary bud. In general, the 1st floral stalk will produce the most mature bud from the 3rd to 5th leaves (Figure 1). If nutrient condition and the maturation of the axillary bud is good, the 2nd most mature axillary bud will develop into the 2nd floral stalk and produce a double-spike plant. If the maturity of two buds are similar, they will spike simultaneously and produce uniform floral stalks that are a sought-after horticultural trait. Double spikes increase flower numbers and therefore increase the price of orchids. 

Besides axillary bud maturation, orchid spiking is temperature dependent; 30 °C retards the transition from the vegetative to the reproductive phases. Actually, the size of AB4 and AB5 are medium large at 30 °C but, so far, no spiking has been found, which may be due to dormancy. That means, besides axillary bud maturation, moth orchids need moderately low temperatures to break dormancy and to trigger spiking. This study shows that 20 °C upregulated *SPK1* and *FT* mRNA in axillary buds and triggered floral spike initiation (Figure 2). Inducer of CBF expression 1 (ICE1) encodes a MYC-like bHLH transcriptional activator and regulates the transcription of *CBF* genes in the cold [20]. Moderately low temperature promotes orchid spiking. However, the temperature regime for orchid spiking is not as cold as some plants need for vernalization [31]. In fact, orchid spiking is a complex process and may have a distinct flowering pathway, and its underlying mechanism requires further extensive study. Moreover, plant hormones may play an important role in orchid spiking, but so far, not much molecular evidence exists in this area. Moderately low temperatures may help plants release ABA, alter hormone homeostasis, or accumulate auxin and cytokinin to enhance cell proliferation. Besides temperature, other environmental factors such as light, nutrient acquisition, etc. might contribute to orchid axillary bud maturation and spike initiation. Of note, protecting orchid from stress conditions can prevent axillary bud degeneration, maximize axillary bud growth and maturation, and finally enhance spiking. Overall, spiking in *Phalaenopsis* orchid is controlled by genetic and environmental cues. 

### 3.2. Identification of a Novel Orchid Spiking Activator, SPK1 

This study identified a novel bHLH TF, SPK1, which contributes to spike induction in moth orchid. Protein structure of SPK1 comprises a conserved helix-loop-helix domain which enables protein–protein interaction. It contains a DNA-binding domain and may bind to the promoter of the downstream target gene. In fact, SPK1 has a conserved basic amino acid Arg that is important for binding DNA (Figure 3b, asterisks). A functional HLH domain is found, and an NLS domain required for nuclear localization is found in SPK1 (Figure 3a). Subcellular localization showed that SPK1 protein localizes to the nucleus (Figure 3d). Overall, SPK1 is a typical bHLH and plays a role as a TF. Phylogenetic tree results showed that SPK1 is evolutionarily conserved in orchid species but less similar to other plant species (Figure 4). That may be because its natural habitat and the spiking/flowering requirement of orchid is unique compared to other plant species. 

### 3.3. SPK1 Controls Meristematic Cell Proliferation

*SPK1* is highly expressed in the meristematic tissues, as revealed in our RT-qPCR and ISH data (Figure 5). It was found that the *SPK1* gene expression pattern in the axillary bud is age dependent. It is highly expressed in young AB2 but gradually reduced in the older axillary buds of AB4 and AB5 (Figure 2a). These results suggest that SPK1 may regulate the early stage of axillary bud development for meristem cell proliferation and organ differentiation. RNA in situ analysis data revealed that *SPK1* mRNA is strongly expressed in the meristem of axillary buds and primordia of the flower bud (Figure 5). Presumably, *SPK1* might mediate flower bud organ differentiation. Moreover, *SPK1* was found highly expressed in the root tip. Our data further showed that *SPK1* is clearly present in the meristematic tissues and may play an essential role in organelle differentiation and cell proliferation. In addition, ISH data also revealed that *SPK1* transcripts are expressed in the vascular bundle of the root, axillary bud, and young floral stalk (Figure 5), indicating that *SPK1* may be involved in long-distance RNA movement in orchids. Plants utilize proteins and RNA as non-cell-autonomous signaling macromolecules to mediate local and long-distance regulation. RNA delivery via the phloem allows flexibility and fine-tuning of developmental programs to ensure newly developing tissues are optimized for performance under different environmental conditions [32]. For example, phloem long-distance trafficking of *GIBBERELLIC ACID*-*INSENSITIVE* RNA regulating leaf development has been reported [32]. 

### 3.4. SPK1 Controls Orchid Spiking 

Gene expression analysis revealed that *SPK1* is expressed in the meristematic tissues of the root and reproductive organs. Under moderately low temperature suitable for spiking, *SPK1* gene is upregulated, but under high temperature, it is downregulated and reduced *SPK1* gene expression (Figure 2a). *SPK1* gene co-expressed with *FT* at 20 °C (Figure 2), implying that SPK1 may be involved in the spiking/ flowering pathway of orchid. Surprisingly, our VIGS experiment showed that downregulation of the *SPK1* gene just slightly suppressed the gene expression level of *FT* (Figure 8f), further suggesting that SPK1 is partly involved in the pathway of FT. Interestingly, in the axillary bud of VIGS-*spk1* plant, the *SPK1* gene is downregulated (Figure 8b), but in turn *bHLH35* is upregulated (Figure 8C). Clearly, bHLH35 and SPK1 act as antagonists and bHLH35 may act as a repressor in the spiking pathway. 

We also found that *CYCB* and *EXPA8* gene expression levels are significantly downregulated in the axillary bud of the VIGS-*spk1* orchid (Figure 8d,e). Presumably, SPK1 may regulate cell cycle genes for cell division. It has been reported that increased expression of cell cycle genes increased mitotic activity and broke dormancy [13]. Moreover, VIGS-*spk1* plants reduce *EXPA8* gene expression, which may reduce cell elongation. Therefore, VIGS of *SPK1* significantly inhibited the 2nd floral spike development (Figure 8g). All orchid plants produced the 1st floral spike (from AB4) whether they were VIGS-*spk1* treated because the orchid plants used in this study were mature and size of the axillary bud was big (Figure 1) before the plants were subjected to VIGS Agroinfiltration. Exposure to 20 °C treatment favors buds breaking dormancy. The axillary bud meristem senses the environmental stimuli and turns on spiking processes. Typically, the VIGS silencing gene occurs at 4 to 6 weeks post-inoculation; therefore, our VIGS on mature orchid plants could inhibit the growth of the young bud and effectively inhibit the 2nd floral spike induction but not inhibit the 1st floral spike. These results indicate that gene hierarchy of SPK1 acts upstream of these known markers associated with flowering, cell cycle, and cell elongation. Therefore, SPK1 TF may regulate several biological functions including axillary bud maturation, spiking time, cell differentiation, and cell elongation, etc. Clearly, orchid spiking is a complex process. Precise coordination between TFs and spatial temporal expression of spiking genes is required for floral spike initiation. 

### 3.5. Coordination among bHLH TFs for Spiking 

Our protein–protein interaction data indicated that SPK1 protein can form a homodimer (Figure 7). Presumably, SPK1 homodimer might modulate downstream genes associated with axillary bud maturation and spiking activation genes. At 20 °C, *SPK1* gene expression level is high (Figure 2a). However, the specific downstream genes that are directly regulated by SPK1 TF remain unknown, whether *CYCB* and *EXPA8* are regulated by SPK1 needs further investigation. It was found that *bHLH35* co-expresses with *SPK1* in the meristematic tissues but that *bHLH35* is upregulated at high temperature and may act as a repressor of spiking (Figure 6). Our in vivo Y2H and BiFC data demonstrated that bHLH35 and SPK1 proteins can form a heterodimer (Figure 7). Presumably, the heterodimer of SPK1/bHLH35 may inhibit the transactivation of downstream spiking gene(s). It has been reported that FD (flowering activator) can interact with TFL1 (flowering repressor) and regulate flowering rhythm [33]. It is believed that precise regulation of the TFs is essential for meristem maintenance and to understand how orchids cope with fluctuation of environments and morphological change. Interaction between SPK1 and bHLH35 might regulate temperature-dependent spike induction in moth orchid. 

### 3.6. Proposed Model for SPK1 Mediates Spiking of Phal. Orchid

**Figure 9:**
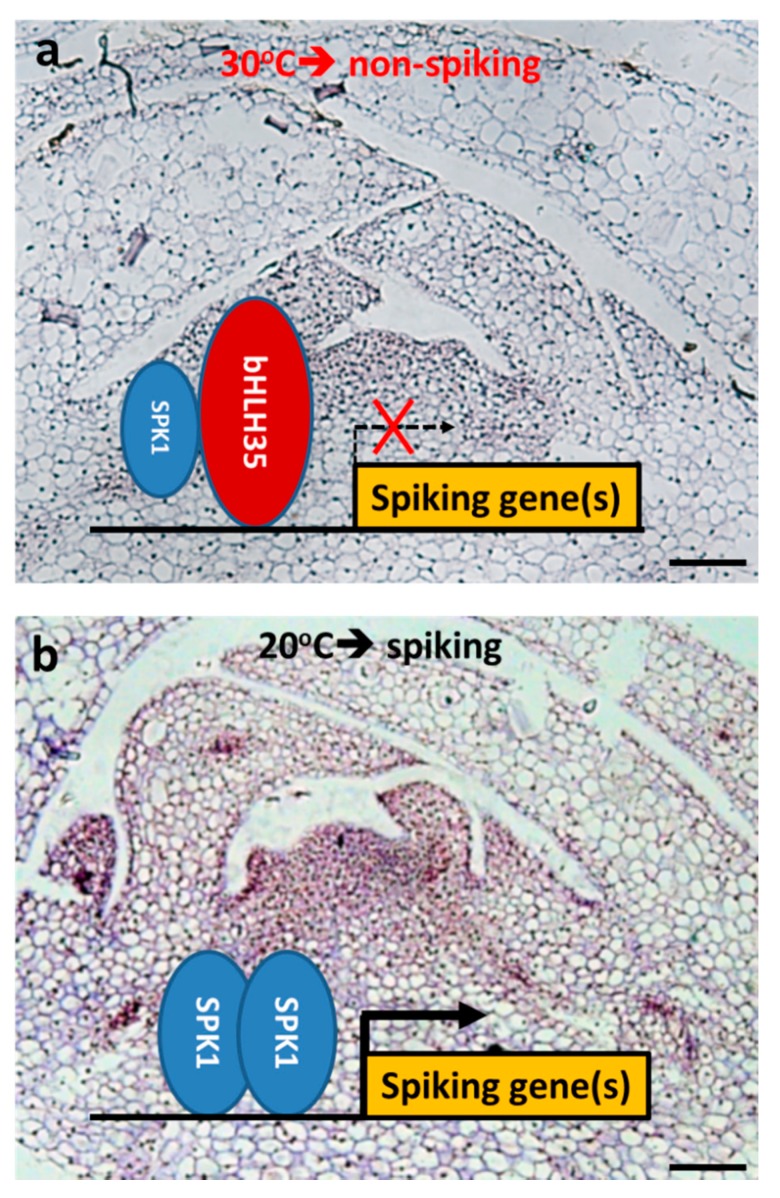
Proposed molecular mechanism underlying SPK1 regulation of axillary bud development and spiking in *Phal*. orchid: (**a**) At 30 °C non-spiking condition, *SPK1* gene is downregulated but *bHLH35* is upregulated. SPK1 and bHLH35 can form a heterodimer and may bind to E-box in the promoter of spiking gene(s) and therefore inhibit the transactivation of downstream spiking gene(s). (**b**) At the 20 °C spiking condition, *SPK1* is highly expressed in the meristem of axillary bud (ISH signal showed purple color). SPK1 forms a homodimer and may bind to E-box in the promoter of spiking gene(s), such as *FT*, *CYCB*, and *EXPA8* for cell division and elongation, and may promote orchid spiking at 20 °C. Bars, 20 μm.

As shown in Figure 9, under 30 °C, although the axillary bud is mature, it is arrested with no spiking (Figure 1). When the axillary bud is exposed to high temperature, *bHLH35* is significantly upregulated (Figure 6b). Therefore, bHLH35 may act as a repressor that inhibits the transactivation of the downstream spiking gene(s), thus inhibiting cell proliferation and causing axillary bud arrested growth with bud size remaining small (Figure 1). We hypothesize that under 30 °C non-spiking condition, bHLH35 might bind to E-box in the promoter of spiking gene(s), inhibit SPK1 homodimer complex binding to the E-box, and fail to transactivate the downstream spiking gene(s). 

However, at moderately low temperature of 20 °C that is good for orchid spiking, *SPK1* is found highly expressed in the meristem of axillary bud (Figure 2a, Figure 5, and Figure 9b), and *FT* mRNA is similarly upregulated in the axillary bud (Figure 2b), further suggesting that the orchid is ready for spiking/flowering. Our VIGS experiments demonstrated that, when VIGS-*spk1* orchid downregulated *SPK1*, it also significantly downregulated the gene expression levels of *CYCB* and *EXPA8* mRNAs (Figure 8), suggesting that gene hierarchy of *SPK1* is located upstream of *CYCB* and *EXPA8*. This study shows two bHLH TFs (SPK1 and bHLH35) that fine-tune axillary bud meristem development and control spike initiation in orchid.

### 3.7. Research Prospects for Control Spiking in Orchid

Double-spike *Phalaenopsis* orchids produce more flowers and have greater market value than those with single spikes. Controlling axillary bud development and maturation is important to meet seasonal market demands. Furthermore, manipulation and control of spiking is essential in the orchid industry for international trading. This study showed that SPK1 regulates growth of orchid meristematic tissues (root tip, axillary bud, young spike, and flower primordium) and cell proliferation and mediates spiking in *Phal.* orchid. It is believed that regulation of spiking is a complex pathway and that discovery of more key genes/TFs expressed in the meristem of axillary bud will extend our understanding of the molecular basis of the spiking pathway in moth orchid. 

## 4. Materials and Methods 

### 4.1. Plant Materials and Growth Conditions

*Phalaenopsis* orchid (*P. aphrodite* subsp. *formosana*) cv. N2K01 orchid plants at the five-leaf stage were purchased from Chain Port Orchid Nursery (Ping Tung, Taiwan). For spike induction, orchid plants were kept in a growth chamber set at a constant 20 °C. For non-spiking treatment, orchids were kept in a 30 °C growth chamber. LED white light was installed in the two growth chambers with a light intensity of 150 mmol^−1^·m^−2^·s^−1^ and 12 h light/12 h dark cycle. After spiking, orchids were then cultivated in the Academia Sinica Biotechnology Center greenhouse in Tainan City in Southern Taiwan. The greenhouse is under a 70% shade curtain to prevent orchids from sunburn, and the temperature was controlled at 24 ± 2 °C. 

### 4.2. Cloning of the SPK1 Gene from Phal. aphrodite

To isolate full-length cDNA, the rapid-amplification of cDNA ends (RACE) technique was performed using the SMART RACE cDNA Amplification Kit (Clontech). Gene-specific primers were designed based on the partial cDNA sequences of *SPK1* (PATC136668); 5′-GGCTCT CCACAATCGTTACCTCTATATCGG-3′ and 5′-GATCTGAAGCCGCTTGCGAGTTTCTTC-3′ primers were used for 5′ and 3′ RACE PCR, respectively. RACE PCR was performed according to the manufacturer’s instructions. 

### 4.3. Phylogenetic Analysis

Protein sequence of SPK1 was blasted in the National Center for Biotechnology Information (NCBI) database. Highly similar sequences were retrieved, and a phylogenetic tree was generated with the neighbor-joining method [34] using Molecular Evolutionary Genetics Analysis, version 10 (MEGA X) software [35] and constructed into a phylogenetic tree. The percentage of replicate trees in which the associated taxa clustered together in the bootstrap test (1000 replicates) are shown next to the branches. The evolutionary distances were computed using the Poisson correction method [36] and are in the units of the number of amino-acid substitutions per site. 

### 4.4. Gene Expression Analysis 

Total RNA was extracted from different tissues of *Phalaenopsis* cv. N2K01 using Trizol reagent (Invitrogen, Carlsbad, CA). Total RNA was DNase treated, and then, 1 mg RNA was reverse-transcribed using M-MLV (Promega) according to the manufacturer’s instructions. All RT-qPCR reactions were performed for 40 cycles with KAPA SYBR FAST qPCR Master Mix 2× (Kapa Biosystems, Woburn, MA, USA). Primers used for PCR are listed in Supplementary Table S1. The real-time RT-PCR reaction was performed in triplicate on the C1000TM Thermal Cycler (Bio-Rad). Relative gene expression level was normalized to the constitutively expressed gene *Ubiquitin* (PATC150470) as a control.

### 4.5. Tissue Section

Orchid tissue samples of axillary buds (AB2 to AB5), top portion of young spikes at 5 cm length and 10 cm length, and 1 cm length of root tip tissue were collected. Paraffin tissue section followed the previous protocol [12]. In brief, tissue samples were fixed in ice-cold 4% Paraformadehyde buffer, dehydrated with serial ethanol, and embedded in a paraffin block, and tissue was sliced vertically to 10 µm thickness.

### 4.6. In Situ Hybridization (ISH) 

ISH protocol was as described previously [37] with some modification. In brief, *SPK1* was cloned using gene specific primers of 5′- CATGAGGAAGAAGGGTTCTATACTTC -3′ and 5′- GCCTT CTCATCATTTGCCTCAT-3’ (213 bp). In situ hybridization of *SPK1* probe was performed at 48 °C with 40 ng of DIG-labeled RNA probe. Anti-DIG is in 1:700 dilution. 

### 4.7. Bimolecular Florescence Complementation Assay 

Full-length cDNAs of SPK1 and bHLH35 were independently introduced into pJET1.2 (Thermo Scientific). The sequence for the N-terminal amino acid residues 1 to 174 of YFP was then in-frame fused to the sequence of the C-terminal region of the tested proteins, while the sequence of the C-terminal amino acid residues 175 to 239 of YFP were in-frame fused to the sequence of the N-terminal end of the proteins. Next, the tested genes were introduced into pSAT5-DEST_CYN1 and pSAT4(A)-DEST_NYN1. Ballistic bombardment-mediated transient transformation was performed as described previously [38]. The bombarded explants were incubated in the dark for 24 h, and florescence images were photographed on a LSM780 Plus ELYRA S.1 confocal microscope with a Plan-Apochromat 403/1.4 oil objective lens (Zeiss, Gina, Germany). 

### 4.8. Yeast-Two Hybrid (Y2H)

The Matchmaker GAL4 two-hybrid system (Clontech,) was used for Y2H assays. The full-length cDNA of *SPK1* was cloned into pGAD-T7 (Clontech). Full-length of *bHLH35* was cloned into pGBK-T7 (Clontech), respectively. Protein interactions in vivo in yeast were carried out according to a previous report [39].

### 4.9. Virus-Induced Gene Silencing (VIGS)

VIGS and *Agrobacterium tumefaciens*-mediated transient expression was performed in *Phalaenopsis* orchids to verify transient loss-of-function assays of SPK1 in orchids. Gene-specific regions of *SPK1* was cloned using the primers referenced in Supplementary Table S1 to obtain 212 bp fragments; it was then cloned into pCymMV-Gateway vectors [30] (Figure S3) and transformed into the agrobacterium strain EHA105. The VIGS vector with the target gene was infiltrated into the 1st and 2nd new established leaves of *Phalaenopsis* orchids in 15 biological replicates. At 6 weeks post-VIGS inoculation, three vector-only control and VIGS-*spk1* plants each were randomly selected and axillary bud samples of AB3 and AB4 were collected, RNA were isolated. Gene expression patterns of *CymMV*, *SPK1*, *EXPA8*, and *CycB* were checked using RT-qPCR. 

### 4.10. Statistical Analysis

Data shown are the means with standard errors of at least 7 independent biological samples. The data were statistically analyzed by Student’s *t*-test. 

## Supplementary Materials

Supplementary materials can be found at https://www.mdpi.com/1422-0067/20/21/5406/s1.

## Abbreviations

AB	Axillary bud
BiFC	Bimolecular florescence complementation
FT	FLOWERING LOCUS T
*Phal.*	*Phalaenopsis aphrodite*
SPK1	Spike Activator 1
RT-qPCR	Quantitative real-time polymerase chain reaction
VIGS	Virus-induced gene silencing
Y2H	Yeast two hybrid
