# Supplementary material for: Spike Activator 1, Encoding a bHLH, Mediates Axillary Bud Development and Spike Initiation in Phalaenopsis aphrodite
Source: Int J Mol Sci. 2019 Oct 30;20(21):5406. doi: 10.3390/ijms20215406 (PMC6862315; doi:10.3390/ijms20215406)
Supplement: Supplementary file 1 [file ijms-20-05406-s001.pdf]

Supplementary data

Lin et al. Spike Activator 1, encoding a bHLH, mediates axillary bud development and spike initiation in *Phalaenopsis aphrodite*

Table S1. Primers used in this work.

Primer Name	Sequence (5' →3')
RACE FL cDNA	
SPK1-5'RACE-R2	GGCTCTCCACAATCGTTACCTCTATATCGG
SPK1-3'RACE-F	GATCTGAAGCCGCTTGCGAGTTTCTTC
	Gene construction
SPK1-SacII-F	TGCCGCGGATGGCTTTAGAAGCCGTGGTC
SPK1-XhoI-R2	TACTCGAGCTGGAAGTATAGAACCCTTCTTCCTC
bHLH35-FLcds-F	ATGGATGCCGACTACGATCACTACT
bHLH35-FLcds-R	AAGGCTCACGCCACTCATT
Y2H- SPK1-EcoRI-F	GCGAATTCATGGCTTTAGAAGCCGTGGTCT
Y2H- SPK1-BamHI-R	TTGGATCCTCAGCTGGAAGTATAGAACCCTTCTTCC
Y2H- bHLH35-EcoRI-F	GCGAATTCATGGATGCCGACTACGATCACTACT
Y2H- bHLH35-BamHI-R	TAGGATCCTCAAAGGCTCACGCCACTCATTGG
VIGS-SPK1-F2	aaaaagcaggctCATGAGGAAGAAGGGTTCTATACTTC
VIGS-SPK1-R2	agaaagctgggtGCCTTCTCATCATTTGCCTCAT
Quantitative RT- PCR	
SPK1-F2	GAAGAGGCGTCGAACCAAGGG
SPK1-R2	GTAAGAAGCAGGCATGAGTGAGCG
PaUbi-F	TGAACTCCATCGCCTTCCTCTTC
PaUbi-R	TGAAGCATGGCATCAATTTC
Pa-CYCB-1f1	TCGTAGCAAGGTTGCTTGTG
Pa-CYCB-1r1	ATGAGCATGGCGCTAATACC
PaFT-F	GAGCAGCCGAGAGTTAAAGTTGG
PaFT-R2	AACTGTCTGTCGGCCTAGTTGATG
bHLH35-F	CATGGAGAGGAACCGCCGC
bHLH35-R	TCCTCCTGATCTATGGCATGATTCC
EXPA8-F	ATGGCAGTTCGGGCAGACCT
EXPA8-R	CGCCTTCCTTCCAACACCAC
CymMV- F	GTCAATGGCCGCGGTACCTA
CymMV-R	GAAATAATCATGGGAGAGCC
In-situ hybridization analysis	
SPK1-F	CATGAGGAAGAAGGGTTCTATACTTC
SPK1-R	GCCTTCTCATCATTTGCCTCAT

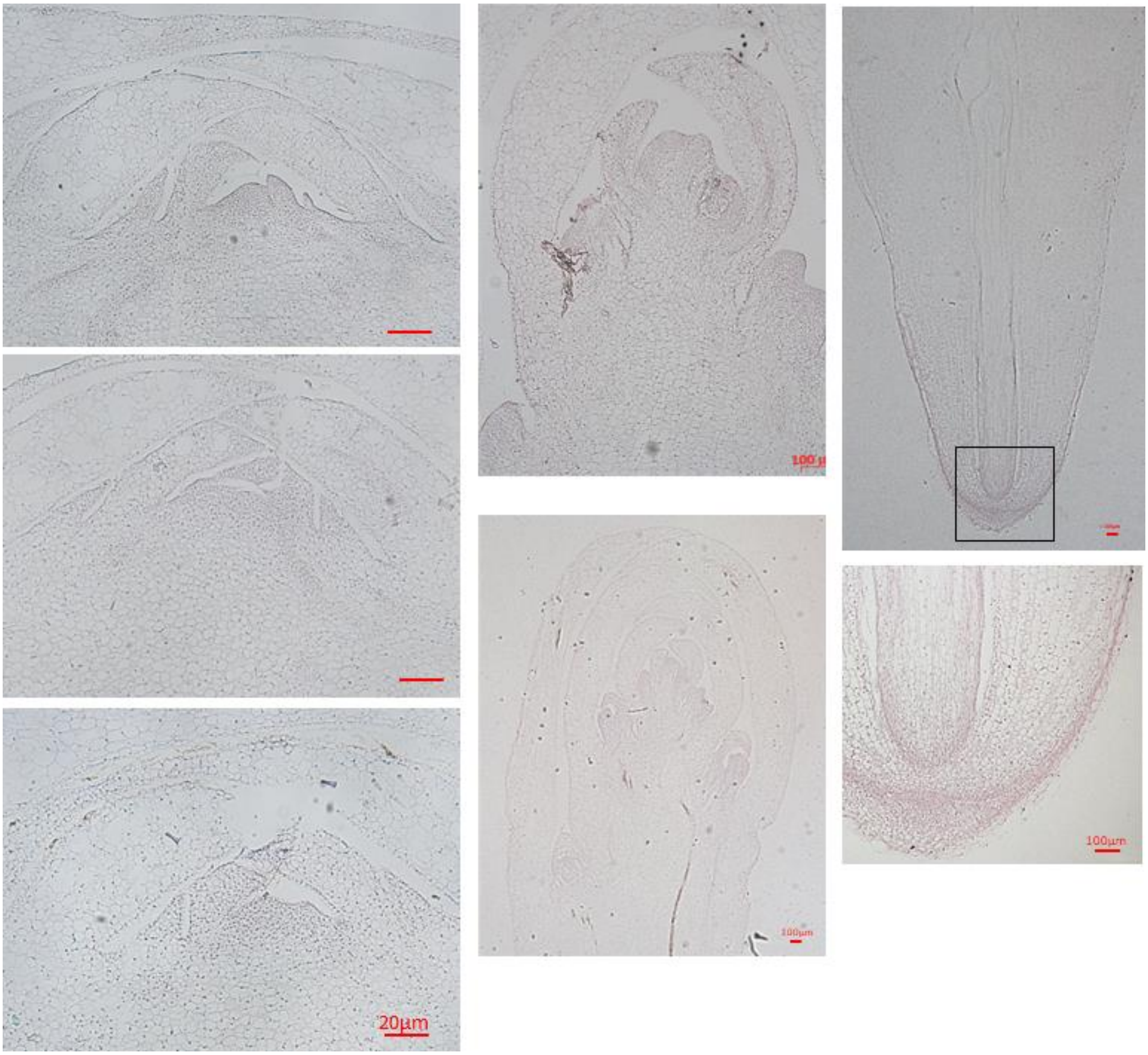

Figure S1. RNA *in situ* hybridization axillary bud tissues using *SPK1*-sense probe.

Fig. S2

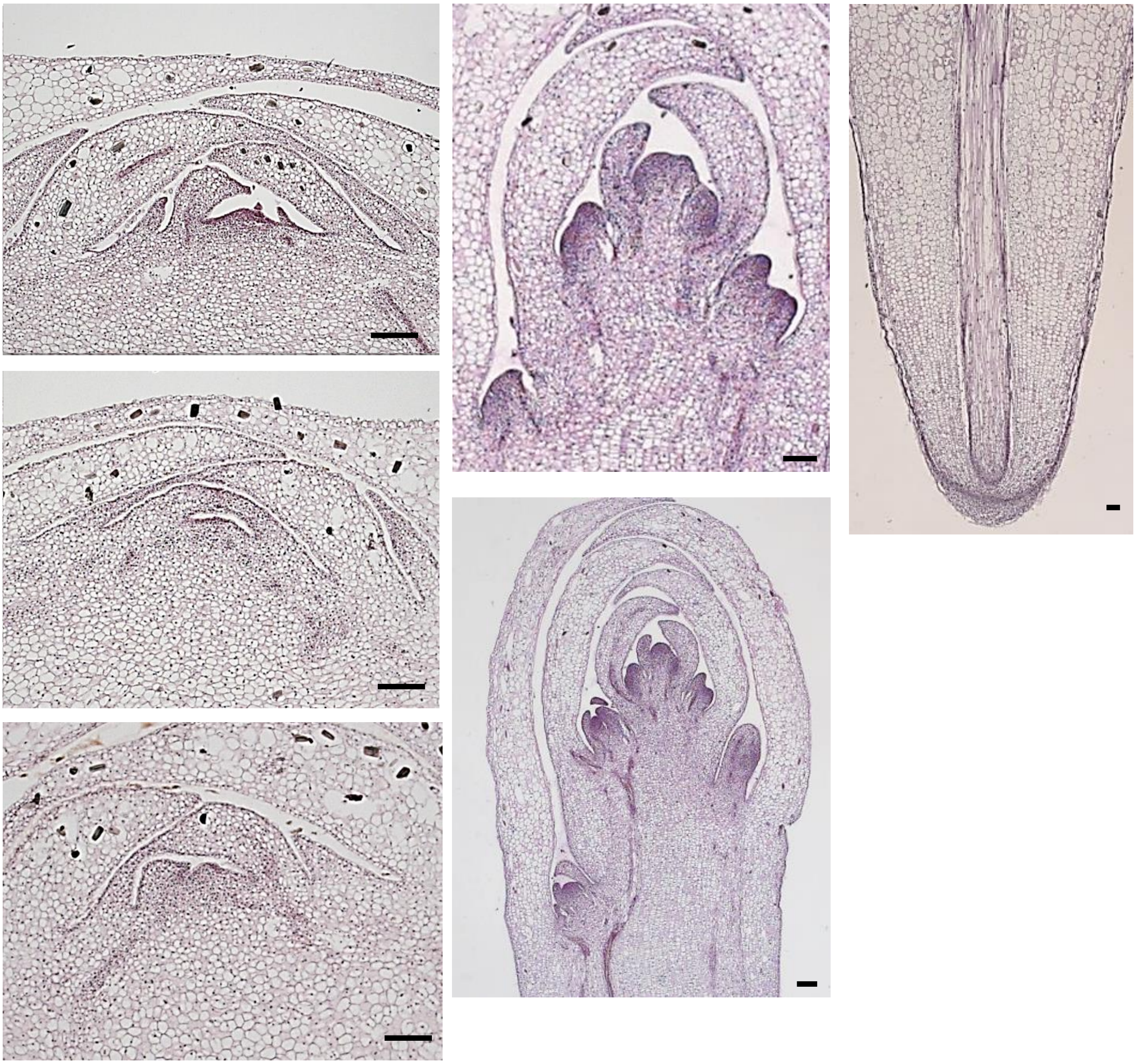

Figure S2. Hematoxylin stained axillary bud tissues for RNA *in situ* hybridization of *SPK1*.

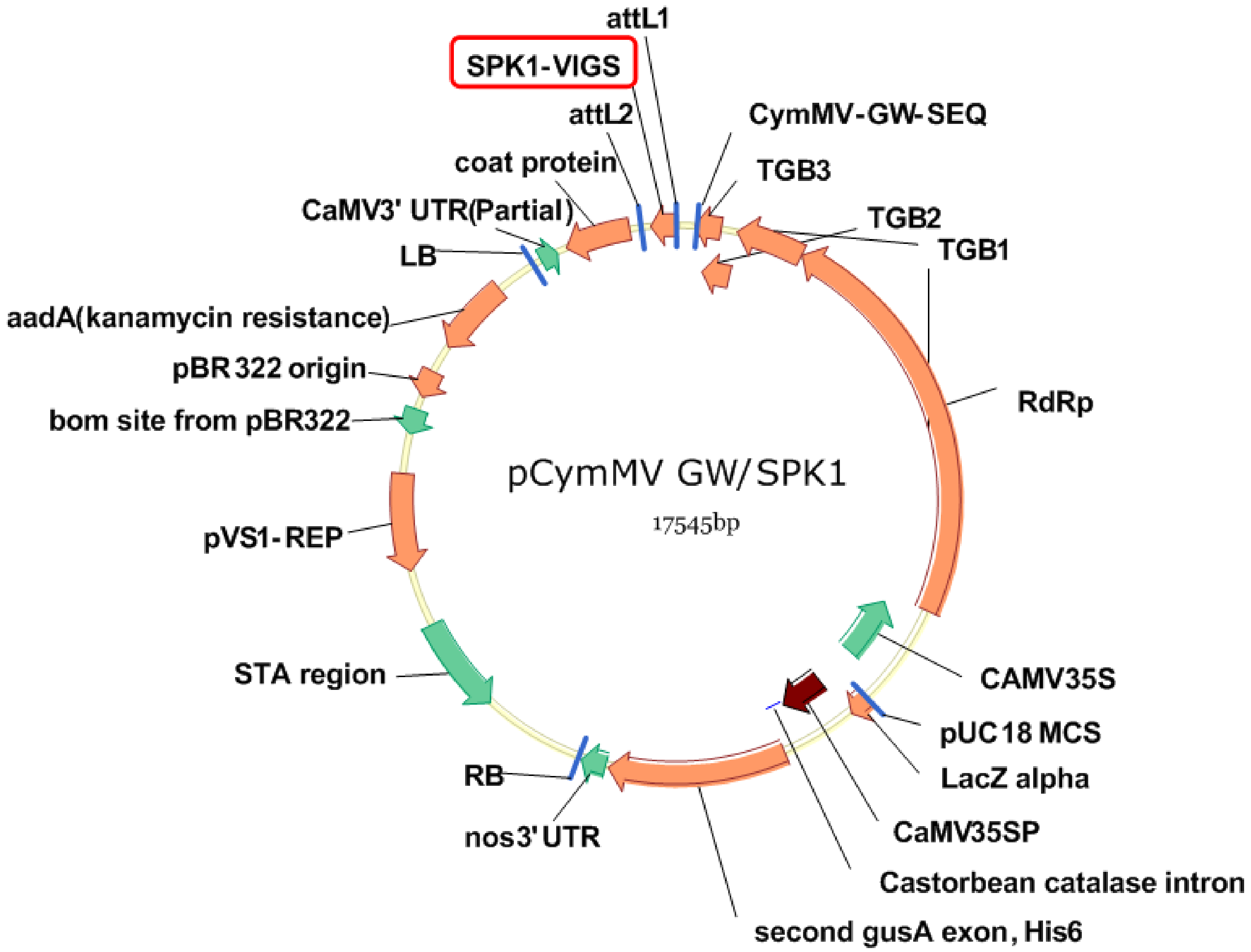

Figure S3. VIGS construction map of *SPK1* used in this study.

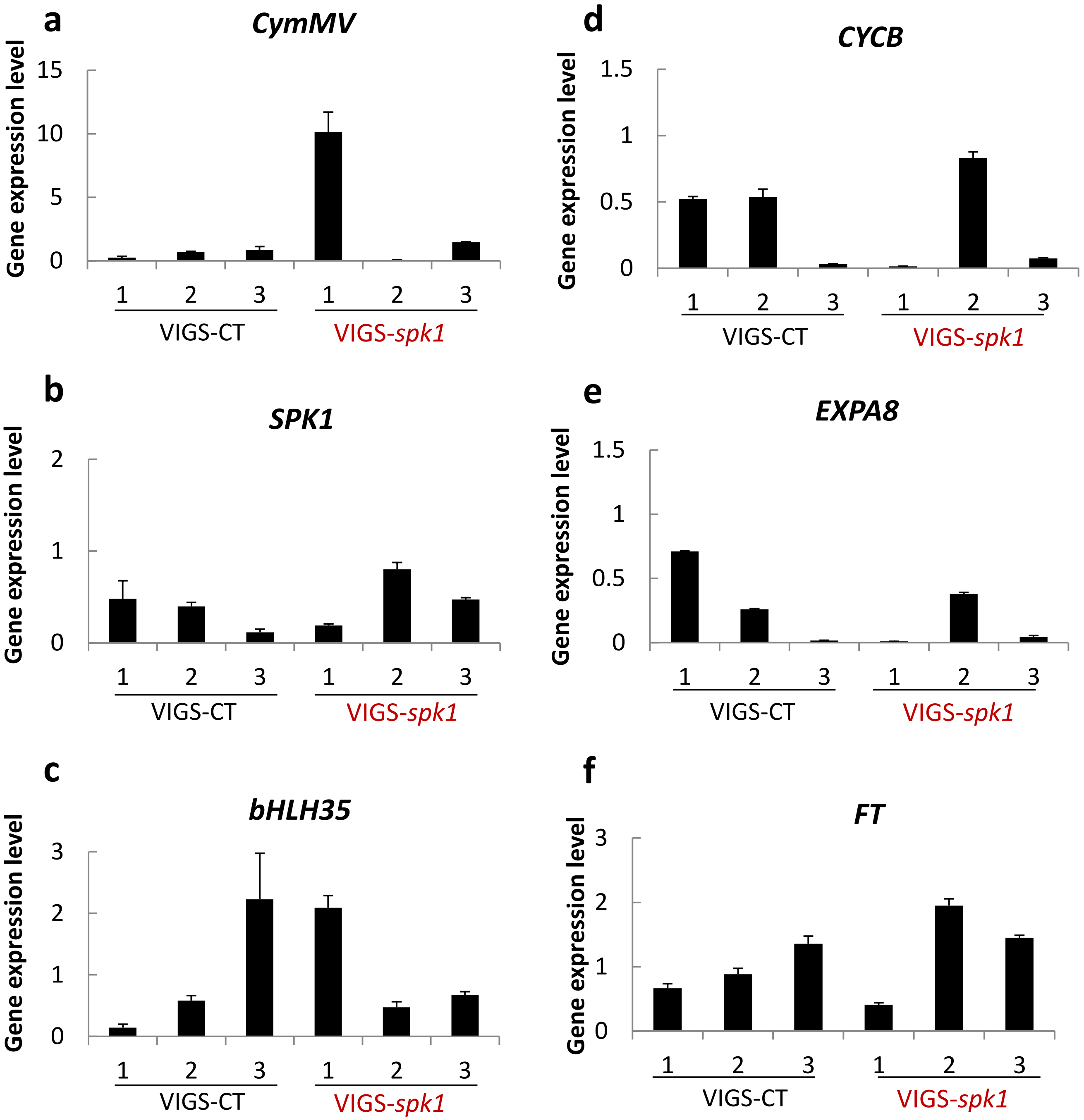

Figure S4. Quantitative RT-PCR showed that VIGS of *SPK1* altered gene expression patterns of *CymMV* (a), *SPK1* (b), *bHLH35* (c), *CYCB* (d), *EXPA8* (e), and *FT* (f) in the 4th axillary bud. Gene expression level is normalized to *Ubiquitin*. Bar: SD of 3 technical replicates.
